# Dissociation Between Linguistic and Nonlinguistic Statistical Learning in Children with Autism

**DOI:** 10.1007/s10803-023-05902-1

**Published:** 2023-02-07

**Authors:** Anqi Hu, Violet Kozloff, Amanda Owen Van Horne, Diane Chugani, Zhenghan Qi

**Affiliations:** 1 Department of Linguistics and Cognitive Science, University of Delaware, 125 E Main St., Newark, DE 19716 USA; 2 Department of Communication Sciences and Disorders, Northwestern University, Evanston, IL USA; 3 Department of Communication Sciences and Disorders, University of Delaware, Newark, DE USA; 4 Department of Communication Sciences and Disorders, Northeastern University, Boston, MA USA; 5 Department of Psychology, Northeastern University, Boston, MA USA

**Keywords:** Autism spectrum disorder, Statistical learning, Language development, Language impairment

## Abstract

Statistical learning (SL), the ability to detect and extract regularities from inputs, is considered a domain-general building block for typical language development. We compared 55 verbal children with autism (ASD, 6–12 years) and 50 typically-developing children in four SL tasks. The ASD group exhibited reduced learning in the linguistic SL tasks (syllable and letter), but showed intact learning for the nonlinguistic SL tasks (tone and image). In the ASD group, better linguistic SL was associated with higher language skills measured by parental report and sentence recall. Therefore, the atypicality of SL in autism is not domain-general but tied to specific processing constraints related to verbal stimuli. Our findings provide a novel perspective for understanding language heterogeneity in autism.

**Supplementary Information:**

The online version contains supplementary material available at 10.1007/s10803-023-05902-1.

## Introduction

Statistical learning (SL), an implicit learning process to detect and extract regularities, plays a fundamental role in the perception and categorization of environmental inputs (Baldwin et al., 2008; Fiser & Aslin, 2002; Saffran et al., 1999). Language contains rich statistical regularities, and SL has been proposed as an important mechanism that underlies typical language acquisition (Romberg & Saffran, 2010). Infants, for example, can segment words in artificial and natural language based on the transitional probabilities between syllables embedded in a continuous stream of speech (Saffran et al., 1996). Children can map individual sounds to their written forms based on the co-occurrences of phonemes and graphemes (Harm & Seidenberg, 1999) and assign lexical stress to written nonwords based on the probabilistic associations between orthography and stress position in a word (Arciuli et al., 2010). SL has also been associated with vocabulary knowledge and reading ability in school-age typically-developing (TD) children (Evans et al., 2009; Qi et al., 2019).

There is growing interest in whether SL plays a role in impaired language (Arciuli & Conway, 2018; Bogaerts et al., 2020b). Children with Autism Spectrum Disorder (ASD) exhibit highly variable language abilities (Kjelgaard & Tager-Flusberg, 2001). Only about 75% of autistic children^1^ are verbal. Among the verbal autistic children, poorer phonological, grammatical, vocabulary, and reading skills are prevalent with large individual variation (Boucher, 2012; Lindgren et al., 2009; McGregor et al., 2012; McIntyre et al., 2017; Tomblin, 2011), which pose challenges for their academic achievement and adversely impact their social-developmental trajectory (Bal et al., 2019; Kim et al., 2018).

There remains a gap in our understanding of the origin of the language variability in autism. In particular, we do not yet know how many autistic children, despite deficits of social cognition, still achieve functional and even advanced language (Naigles & Chin, 2015). It has been postulated that language acquisition in this population capitalizes on implicit learning, such as SL, which can occur as a byproduct of mere exposure with minimal social interaction (Naigles, 2013, 2016). The presence of language delay/ impairment in autistic children might be a consequence of atypical learning mechanisms including SL (Walenski et al., 2006). Yet, mixed findings regarding the existence of SL deficits have been reported in individuals with autism (see Obeid et al., 2016 for meta-analysis; Arciuli, 2017; Saffran, 2018 for reviews). While some studies showed intact SL (e.g., Barnes et al., 2008; Brown et al., 2010; Haebig et al., 2017; Mayo & Eigsti, 2012; Roser et al., 2015), others have shown atypical SL. Atypical SL is often manifested as slower detection of patterns, lowered accuracies in pattern retrieval, or reduced neural activation to patterns compared to controls (e.g., Arciuli & Paul, 2012; Jeste et al., 2015; Scott-Van Zeeland et al., 2010). SL has also been related to verbal IQ and receptive vocabulary in autism (Haebig et al., 2017; Jeste et al., 2015; Jones et al., 2018), while null results using similar assessments have also been documented (Foti et al., 2014; Jones et al., 2018; Mayo & Eigsti, 2012). A few common methodological limitations across this body of literature may have contributed to the inconclusive findings: the use of a single task to describe SL abilities, the assumption of developmental invariance, and the limitation of reflection-based (aka offline) learning measures.

SL has been traditionally treated as a uniform skill and represented by tasks in a single sensory modality (visual or auditory) or domain (linguistic or nonlinguistic). As a result, this approach may have contributed to the lack of consensus as to whether SL is impaired in autistic individuals. For example, studies using nonlinguistic visual stimuli (e.g., abstract geometric shapes) found no group differences in SL between ASD and TD groups (Barnes et al., 2008; Brown et al., 2010; Nemeth et al., 2010). However, a study using linguistic visual stimuli (written pseudo-words) reported reduced sensitivity to orthographic regularities in adolescents with ASD (Arciuli & Paul, 2012). In addition, cognitive neuroscience studies investigating SL in children with ASD reported reduced neural responses to statistical patterns embedded in linguistic auditory sequences (Scott-Van Zeeland et al., 2010) and reduced electrophysiological responses to visual sequences (Jeste et al., 2015), whereas null findings have been found in behavioral studies using similar stimuli (Brown et al., 2010; Mayo & Eigsti, 2012). Rather than being inconsistent, these findings raise the possibility that processing capacities for different types of stimuli might cast different degrees of constraints on SL performance in individuals with ASD (see Arciuli, 2017 for a review). Indeed, recent findings in the general population suggested SL performance can be constrained by processing capacity, which varies across sensory modalities. For example, Conway and Christiansen found that neurotypical adults were better at learning temporal statistical patterns embedded in the auditory modality (pure tones) than those embedded in the visual modality (colored shapes) (Conway & Christiansen, 2009). Furthermore, individual performance in auditory linguistic SL tasks where patterns were embedded in streams of syllables showed no correlation with performance in nonlinguistic tasks (Arnon, 2020; Siegelman & Frost, 2015), suggesting learning ability varies not only across, but also within, individuals. Separate processes of learning might underlie SL tasks with different types of stimuli. Taken together, these findings raise concerns about the validity of operationalizing SL using a single task.

Limited understanding of the developmental trajectory of SL may also contribute to the mixed pattern of group differences. Using an auditory linguistic SL task, Saffran et al. exposed participants to a continuous stream of artificial speech stimuli made up of trisyllabic nonsense words (e.g., *ba-bu-pu*). In the speech stream, the syllables within the trisyllabic words always co-occur together, while syllables across word boundaries co-occur with a much lower transitional probability. They found that 6- and 7-year-old TD children performed similarly as adults and were able to learn the trisyllabic words and distinguish them from nonsense words that never occurred in the artificial speech (Saffran et al., 1997). Recent reports demonstrated consistent patterns that children 6.5 to 12 showed better than chance-level SL performance in the linguistic domain, with no correlation between age and performance (Raviv & Arnon, 2018). In contrast, SL in the nonlinguistic domain improved with age between 5 to 12 years-old in TD children (Arciuli & Simpson, 2011; Raviv & Arnon, 2018; Shufaniya & Arnon, 2018). In children with ASD, understanding of how SL changes across development is even more limited. One study reveals an age effect on SL across ASD and TD (Jeste et al., 2015), while others reported a lack of age effect in the ASD group (Jones et al., 2018; Mayo & Eigsti, 2012). These mixed findings may reflect true differences in the developmental trajectories between different types of SL in children with ASD. Yet, substantial differences in task designs and age ranges necessitate a systematic investigation on the development of SL using a paradigm that combines similar tasks across sensory modalities and linguistic vs. nonlinguistic domains. Furthermore, how SL performance in autistic children compare to TD children across development remains unclear. Recent findings in adults support a relationship between prior linguistic experience and SL learning outcomes. Prior language experience has been shown to facilitate learners’ SL ability in the linguistic domain when items to be learned are similar to or consistent with one’s familiar natural language (Elazar et al., 2022; Perfors & Kidd, 2022; Siegelman et al., 2018a; Trecca et al., 2019). Moreover, adults’ ability to learn trisyllabic nonsense words in artificial speech has been associated with greater sensitivity to high-frequency trigrams in natural language (Isbilen et al., 2022). Ample evidence in TD infants has also shown that prior experience affects SL learning outcomes (see Saffran & Kirkham, 2018 for a review). Infants can learn combinatorial patterns of familiar stimuli, such as pictures of dogs and cats or syllables, but not when the stimuli were unfamiliar nonlinguistic tones or timbres (Marcus et al., 2007; Saffran et al., 2007). When infants are exposed to individual nonsense words prior to listening to an artificial speech stream, the prior experience with individual nonsense words only facilitated SL when the individual nonsense words matched the patterns in the artificial speech stream, but not when they were mismatched (Lew-Williams & Saffran, 2012; Saffran & Thiessen, 2003). As the amount of linguistic experience increases with age in children, familiarity with linguistic inputs and language proficiency are expected to increase across development, which might in turn facilitate SL in the linguistic domain. Yet, autistic children’s difficulties in processing and using language, in turn, may result in reduced language experience that leads to less robust SL from linguistic inputs. Linguistic SL skills thus may show distinct developmental trajectories between TD and autistic children. Yet, the hypothesis that linguistic SL develops differently in TD and autistic children has not received empirical support.

Finally, recent psychometric scrutiny of SL performance measures advocates for direct measures of the learning process. The traditional two-alternative forced choice (2-AFC) task, despite being a robust measure to determine whether learning occurred at the group level, does not produce high test–retest reliability within individuals, especially in developmental research (Arnon, 2020). This task requires children to explicitly reflect on the learned information after extensive repetitions of the stimuli and therefore may only reflect perceptual preference as a learning outcome (Siegelman et al., 2017). In contrast, reaction time during the familiarization phase of a SL task measures automaticity during the learning process (Batterink, 2017; Turk-Browne et al., 2005), while exerting relatively little cognitive demand on participants. Reaction time was found to be related to post-learning 2AFC accuracy (Qi et al., 2019; Siegelman et al., 2018b), yielding high test–retest reliability in adults (Siegelman et al., 2018b) and modest-to-high split-half reliability in school-aged children (Zinszer et al., 2022). Therefore, a combination of reflection-based and process-based measures of SL are necessary for us to compare both the learning outcomes and the learning rate between groups.

These concerns have limited our understanding of SL and its relationship with language in both typical and autistic children. In TD children, whether such a relationship can be observed empirically depends on the type of SL tasks, the age range of participants, and the construct of interest in the language domain (e.g., Bogaerts et al., 2020a; Kidd & Arciuli, 2016; Lany et al., 2018; Qi et al., 2019). In children with ASD, similarly divergent findings have been reported in a much smaller body of literature. Both a positive correlation and null findings between SL and vocabulary abilities were found in children with ASD (Haebig et al., 2017; Mayo & Eigsti, 2012; Scott-Van Zeeland et al., 2010). Therefore, a systematic examination of the associations between language and SL across domains and modalities is needed for understanding the role of SL in the language development of autism.

### Current Study

The current study aims to understand the relationship between language and SL by systematically comparing SL profiles in school-aged children with ASD to TD children across modalities and domains, while addressing several common methodological limitations in previous literature. Four child-friendly web-based SL tasks (Schneider et al., 2020), each containing auditory linguistic (Syllable), auditory nonlinguistic (Tone), visual linguistic (Letter), or visual nonlinguistic (Image) stimuli, adopt a similar design of the familiarization and test phases as seen in commonly used triplet-learning paradigms (Arciuli & Simpson, 2011; Saffran et al., 1999). We assess SL performance through both the acceleration of reaction time (RT) during the familiarization phase and through offline accuracy in the two-alternative forced choice (2-AFC) test phase.

Due to the atypical language profiles often observed in children with ASD, we hypothesize a particular difficulty with linguistic SL in the ASD group. Furthermore, to investigate the relationships between SL and language development, we examine how SL performance varies across parental-rated language levels, across individual language skills measured by Redmond Sentence Recall, and across developmental stages. We hypothesize that only linguistic SL performance will be associated with language skills. The diagnostic group effect will become more evident in older children only for linguistic SL but not for nonlinguistic SL, as children’s familiarity towards syllables and letters grow with their language experiences throughout the school years, while their familiarity towards image and tone sequences in the experiment should not vary across age.

## Methods

### Participants

Fifty-five children with ASD (6.17–12.17 years; 10 girls), recruited from the Simons Foundation Powering Autism Research for Knowledge database (SPARK Consortium, Feliciano et al., 2018) and 50 age-matched TD children (6–12.58 years; 28 girls), recruited nationally through online advertisement (e.g., Facebook and childrenhelpingscience.com), participated in this web-based study. Children with ASD were recruited by SPARK from nationwide clinical sites and were screened based on parent- or caregiver-reported professional diagnoses, age of diagnoses, and questionnaires of autistic symptoms (The SPARK Consortium, Feliciano et al., 2018). The caregiver-reported autism diagnoses in SPARK were further validated by children’s medical records (Fombonne et al., 2022). Professional diagnoses of ASD were obtained from a clinical psychologist (*N* = 10), a team of health care professionals (*N* = 8), a team of professionals in a school system (*N* = 3), a specialty medical doctor (*N* = 3), a pediatrician or primary care doctor (*N* = 2), or from more than one of these sources (*N* = 28).^2^ The diagnoses were further confirmed using the Social Communication Questionnaire (SCQ > 15) (Rutter et al., 2003) and Repetitive Behavior Scale—Revised (RBS-R) (Bodfish et al., 1999) (Table 1). All children were native speakers of English with normal or corrected-to-normal vision, no history of hearing loss, recent ear infections, mutism, or (non-verbal) learning disabilities based on parental questionnaires. TD children were neurotypical and had no history of speech or language impairments. For those with returned questionnaires (*N* = 40), no TD participant had an SCQ-Current score higher than 15. As gender is not matched between diagnostic groups, gender (female vs. male) was entered as a covariable for all analyses investigating group differences.

**Table 1:** Demographics and language assessment scores of participants

	Characteristics	TD	ASD	Group difference
Full sample	N (# of Girls)	50 (28)	55 (10)	$\chi$ ^2^ = 14.62, *p* < 0.001
	Mean Age (SD)	8.63 (1.89)	8.28 (1.20)	*t*(81.49) = 1.12, *p* = 0.27
	Mean SCQ (SD)	3.00 (2.41)	19.38 (6.91)	*t*(60.14) = -15.34, *p* < 0.001
	Mean RBS-R (SD)	–	32.09 (22.65)	
	At/above-age vs. Below-age Language Level	–	20 vs. 26	
Sample with language assessment	N (# of Girls)	34 (19)	33 (3)	$\chi$ ^2^ = 14.57, *p* < 0.001
	Mean Age (SD)	8.22 (1.62)	8.5 (1.31)	*t*(62.97) = − 0.79, *p* = 0.43
	Mean Sentence Recall Raw Score (SD)	27.26 (4.61)	19.91 (8.18)	*t*(50.20) = 4.52, *p* < 0.001
	Mean Sentence Recall Standard Score (SD)	110.11 (13.67)	80.50 (23.78)	*t*(43.39) = 5.69, *p* < 0.001
	Mean SCQ (SD)	2.96 (2.50)	20.25 (6.96)	*t*(33.87) = − 12.37, *p* < 0.001
	Mean RBS-R (SD)	–	37.39 (25.17)	

*SCQ* Social Communication Questionnaire. SPARK families completed the Lifetime form. We followed up with parents of TD children with the Current form. Seven children with ASD and ten TD children were missing SCQ scores. *RBS-R* Repetitive Behavior Scale-Revised. Eight children with ASD were missing RBS-R scores

### Language Assessment

The overall language levels of children with ASD were rated by parents. Parents selected the language levels based on four categories: significantly-below, slightly-below, at, or above age levels. Twenty children with ASD had at or above age language levels, while 26 were slightly or significantly-below age.^3^

We also offered all child participants an opportunity to complete a standardized language assessment, the Redmond Sentence Recall Task (Redmond, 2005), as a measure of language skills (see procedure below). Because this task was adapted and administered as a web-based task (de Leeuw, 2015), the technical challenges of audio-recording on web-browsers have limited our data collection capacity in the early phase of this study. Therefore, this task was made optional for families to complete. Thirty-three of 55 children with ASD and 34 of 50 age-matched TD children completed the task (Table 1).

The web-based Sentence Recall task adapted from Redmond (Redmond, 2005) was used to assess children’s ability to repeat sentences that contain regular past tense forms and past participle forms. We chose this task due to its high sensitivity and specificity for detecting language impairment in children from ages 5 to 9.5 (Archibald & Joanisse, 2009; Redmond, 2005). Children were instructed to repeat 16 sentences each composed of ten words with an equal number of active and passive sentences after a practice sentence. Identical to the original in-person task, children were told that they will listen to some sentences, and they should repeat exactly what the speaker says. Sentence stimuli were pre-recorded by a female native speaker of English. Children’s verbal responses were recorded with their own computer microphone at home. Parents then were instructed to upload the recordings to a secure cloud-based drive. Because norming data are only available for children between 5 and 9.5 years old, we obtained raw scores for all participants and standard scores for children less than 9.5 years (27 children with ASD and 28 TD children). The raw scores and age-normed standard scores are significantly higher in the TD than the ASD group (Table 1).

Within the group of children with ASD, those whose language levels were reported at or above age level by their parents had significantly higher raw and age-normed standard scores on the Sentence Recall task than those whose language levels were below age level^4^ (*p*’s < 0.04), lending credence to the use of the parental-report measure to characterize children’s language groupings.

### Materials and Design of the SL Tasks

#### Overall Design

The detailed design and procedures for the SL tasks have been previously described in Schneider et al. (2020). We examined children’s SL performance using image (visual-nonlinguistic), letter (visual-linguistic), tone (auditory-nonlinguistic), and syllable (auditory-linguistic) stimuli. For each SL task, a familiarization phase, in which children performed a target detection task for about 5 min, was immediately followed by a test phase, in which a 2-AFC test was given. All experimental data are available on https://osf.io/4k7mx/?view_only=57a10c4f834f4113874bff27d87cedfd.

#### Stimuli

**Fig. 1:**
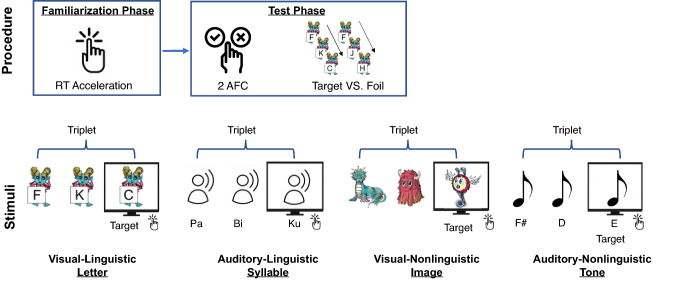
Overall procedure (top row) and the stimuli examples (bottom row) of the SL tasks

In the familiarization phase, stimuli were presented in a continuous stream according to an embedded pattern of four unique triplets (see Fig. 1). In the two visual tasks, 12 unique alien images (Image Task) and 12 images of an alien cartoon character holding a capital letter (Letter Task) formed four target triplets. Each of the target triplets was repeated 24 times for a total of 96 triplets. Each image was presented one at a time at the center of the screen for 800 ms with 200 ms of inter-stimulus interval (stimulus onset asynchrony (SOA) = 1000 ms), lasting 4 min 48 s. In the two auditory tasks, 12 unique monotones of the same duration (328 ms) (Tone Task) and 12 unique syllables (Mean Duration = 350 ms, *SD* = 40 ms) (Syllable Task) formed four target triplets. Each triplet was repeated 48 times for a total of 192 triplets. The SOA was 480 ms, with the familiarization phase lasting 4 min and 36 s. Presentation speed was faster in the auditory than visual tasks due to differences in perceptual preference (Conway & Christiansen, 2009).

Each test phase was composed of 32 2AFC questions. For each question, two options were included: a target triplet from the familiarization phase and a foil triplet that was novel to the participant. Foil triplets were constructed so that the relative position of each image in the foil triplet was the same as the target triplet. The test phase consisted of 32 (4 target triplets × 4 foil triplets × 2 repetitions) randomly ordered trials. The images and sounds within each triplet were presented one at a time at the same presentation rate as the familiarization phase with a 1000 ms pause between the target and the foil triplets.

### SL Task Procedure

The order of the four SL tasks was counterbalanced across participants. Children completed all tasks at home via a secure link. Parents were given step-by-step written instructions and visualizations on how to use the link. Upon opening the study link, a web page with a column of six buttons corresponding to a volume check task, the four SL tasks, and the Sentence Recall task were presented. Parents were instructed to assist children with adjusting computer volume and opening the individual task links while letting their children complete the tasks on their own in a quiet environment. Parents could also check off each task upon its completion and leave comments at the end of each task to reflect their children’s performance. The instructions for children started with a volume-check task, during which a “Happy Birthday” song was played on the frontpage. Children were asked to answer two Yes/No questions regarding what the song was (was it *Baa Baa, Black Sheep*? and was it *Happy Birthday*?). Feedback was given to make sure children could hear their computer audio before starting the SL tasks. In the SL tasks, instructions were given before each task regarding whether the task required watching or listening. Children were encouraged to complete each of the 10-min SL task in one sitting, but they could take breaks in between tasks. All instructions were embedded on the webpage and were pre-recorded by a female native English speaker. Children were given one week to complete all SL tasks. The order of the SL tasks was randomized across individuals. Six children with ASD and 7 TD children chose to conclude the study after completing three out of the four tasks (See Table S1 for the number of participants in each task).

During the familiarization phase, children completed a target detection task. Participants were shown a target stimulus during the instructions and were told to press the space bar as soon as they saw or heard the same target during the familiarization phase. The target stimulus tracked by participants was the third stimulus of one of the triplets, randomly assigned for each SL task for each participant. Practice trials were provided before the familiarization phase and no explicit instructions were provided about the presence of the triplets. During the test phase, participants were instructed to choose whether a target or a foil triplet was more similar to what they saw or heard in the familiarization phase. Each test trial ended with a response and no feedback was provided.

### Analyses

Statistical learning is measured both during the familiarization phase and at the test phase. During the exposure phase, RT for the target stimulus is treated as an index of online learning. If children can update their representations of the incoming inputs and compute statistical regularities as they track the statistics gradually, reduced RT should be observed during the familiarization phase. To include both anticipatory responses and delayed responses, we accepted key presses in the time window of one stimulus before and one stimulus after the target stimulus (Auditory task: − 480 ms to + 960 ms; Visual task − 1000 ms to + 2000 ms). Outliers were removed based on the number of hits (keypresses on target stimuli) and the sensitivity to target stimuli versus false alarms (keypresses on non-target). Children who had fewer than 6 valid keypresses in a task were removed from the RT analysis for that task. *A’*, a measure of the sensitivity for correctly detecting a stimulus based on hit and false alarm rates,^5^ was calculated for each individual and task (Aaronson & Watts, 1987; Grier, 1971; Pallier, 2002). Extreme outliers who scored lower than 3 *SD*s of the mean *A’* of the group were removed. These procedures removed 3.61% of data in the RT analyses (See Table S1 for *N*s). An additional child with ASD was removed from the RT analysis for the Syllable task due to technical issues of the host website. The mean number of valid responses (hits) and *A’* were not significantly different between TD and ASD groups for Syllable, Image, and Tone SL, and were marginally different for Letter SL task (Table S2). RTs for each participant and each task were transformed into *z* scores before the calculation of RT slope over the course of the familiarization phase so that baseline RT differences between participants, domains, and modalities could be controlled for. For each participant and task, we estimated the coefficient of the trial effect on RTs using linear regression models with the *z*-normed RTs as the dependent variable and the target trial order (visual: 1 to 24; auditory: 1 to 48) as the independent variable. A negative RT slope indicated accelerated target detection and indexed online SL. This approach of measuring online learning has been validated in our previous work in adult learners who accelerated more quickly in their responses to target stimuli in structured sequences similar to the ones used here than in random sequences where no triplets were formed and the same stimuli were displayed in a random order (Kozloff et al., 2018; Schneider et al., 2020). RT acceleration was also observed in children using a tablet to respond to target stimuli at the final position of a triplet, but not for target stimuli at the start position of a triplet (Zinszer et al., 2022). In the test phase, SL learning outcomes were measured both by binomial accuracy (0 vs. 1) from each test trial and percentage of correct trials across all 32 test trials for each task and participant.

Because online and offline learning measures reflect different aspects of SL, we computed a composite SL score for each task to capture a comprehensive assessment of children’s SL ability. We defined the composite score for each individual for each SL task as the average of *z*-normed RT slope (with reversed sign so that quicker learning is corresponding to more positive value) and *z*-normed 2AFC accuracy, with norms computed for the whole sample. We further calculated the linguistic and nonlinguistic composite scores by averaging across the four *z*-normed SL measures (2 RT slopes and 2 accuracy scores) within each domain.

## Results

### SL Task Performance Within Each Group

#### Online Learning During the Familiarization Phase

**Fig. 2:**
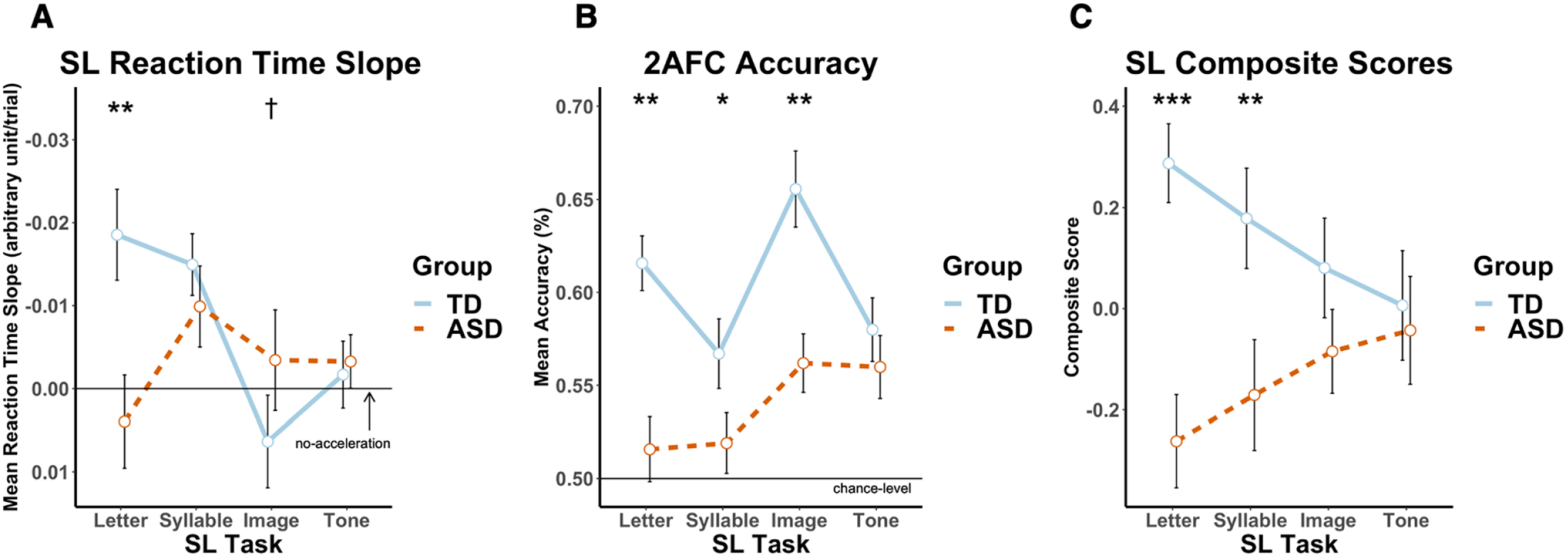
**a** Mean RT slope during the familiarization phase in TD children and children with autism spectrum disorder (ASD) compared against zero. Zero indicates no acceleration. More negative slopes (plotted up) indicate faster acceleration in target detection. **b** Mean accuracy in the test phase (chance-level = 50%). **c** Mean composite statistical learning (SL) scores combining RT slope and accuracy. Higher composite scores indicate better SL performance. Stars marked group differences. ***p < 0.001, **p < 0.01, *p < 0.05, and ^†^*p* < 0.1. The error bars represent within-group standard error (Morey, 2008)

Online SL was defined by a negative RT slope compared against zero, as tested by one-tailed one-sample *t*-tests. The RT slope in TD children was significantly below zero in the linguistic SL tasks (Letter and Syllable; *p*’s < 0.002) but not in the nonlinguistic tasks (Image and Tone; *p*’s > 0.16). The RT slope in children with ASD was only significantly below zero in the Syllable SL task (*p* = 0.02) and marginally below zero in the Tone SL task (*p* = 0.09) (Fig. 2a; Table S3).

#### Offline Learning from the 2AFC Task

Successful offline learning was defined by higher than 50% chance-level accuracy tested by one-tailed one-sample *t*-tests. TD children performed significantly better than chance-level in all tasks (*p*’s < 0.001) (Fig. 2b; Table S3). Children with ASD identified the target triplets significantly above chance-level in the nonlinguistic tasks (Image and Tone; *p*’s < 0.002), and marginally above chance-level in Syllable (*p* = 0.06), but not in the Letter task (*p* = 0.22).

### Group Differences in SL Performance

#### Online Learning During the Familiarization Phase

To examine the differences in online SL between the TD and ASD groups, we analyzed the fixed effects of diagnostic group (ASD vs. TD), domain (nonlinguistic vs. linguistic), modality (auditory vs. visual), target trial order (1 to 24 in visual SL tasks; 1 to 48 in auditory SL tasks), and their interactions on RT using a mixed-effects linear regression model (Barr et al., 2013). The model included random intercepts for participants, and random by-participant slopes of domain and modality. The fixed effect parameters from this model were summarized in Table S4. There was a significant effect of trial order: participants responded more quickly in later than earlier trials (*b* =  − 0.006, *SE* = 0.001, *t* =  − 4.69, *p* < 0.001). We also found a significant domain by trial order interaction (*b* =  − 0.01, *SE* = 0.002, *t* =  − 3.94, *p* < 0.001***), indicating a faster RT acceleration in the linguistic than the nonlinguistic tasks. Furthermore, this advantage was more evident in the TD group than in the ASD group as revealed by a significant three-way interaction between diagnostic group, domain, and trial order (*b* =  − 0.02, *SE* = 0.005, *t* =  − 3.65, *p* < 0.001***). In other words, the ASD group was specifically slower in online learning for the linguistic SL tasks, compared to the TD group. We also observed a significant four-way interaction between diagnostic group, domain, modality, and target index (*b* =  − 0.02, *SE* = 0.01, *t* =  − 2.43, *p* = 0.02), reflected as a larger TD advantage in Letter SL than Syllable SL task in the linguistic domain. Although in the nonlinguistic domain, the ASD group showed numerically quicker RT acceleration than the TD group, post-hoc group comparisons within each SL task using one-tailed *t*-tests confirmed that the group differences were not significant. Supplementary analyses including hits and *A’* of the familiarization phase as covariables did not change the main results.

#### Offline Learning from the 2AFC Task

To examine learning outcomes in the test phase, we analyzed the fixed effects of diagnostic group, domain, and modality on the accuracy from the test phase using mixed-effects logistic regression. The dependent variable was the trial-by-trial binomial accuracy data. The model included random intercepts for participants and test items, and random by-participant slopes of domain and modality (Table S5). We found a significant group effect (*b* = 0.32, *SE* = 0.09, *z* = 3.45, *p* < 0.001) with the TD group performing better than the ASD group. There was also a significant effect of domain with higher accuracy in the nonlinguistic than the linguistic domain (*b* =  − 0.16, *SE* = 0.05, *z* =  − 3.10, *p* = 0.002) and a significant effect of modality with higher accuracy in the visual than the auditory modality when collapsed across groups (*b* = 0.19, *SE* = 0.07, *z* = 2.60, *p* = 0.009). Furthermore, a significant interaction was found between group and modality (*b* = 0.34, *SE* = 0.14, *z* = 2.34, *p* = 0.02), indicating the TD group’s advantage is more evident in the visual than auditory modality. This group by modality interaction was mainly driven by the lack of group differences in the Tone SL task as confirmed by post-hoc comparisons. The TD group learned significantly better than the ASD group in the Letter, Syllable, and Image (*p*’s < 0.02), but not in the Tone SL task (*p* = 0.21).

#### Group Differences in SL Composite Score

We also compared the two groups in the composite score which is a more comprehensive measure for both learning outcomes and learning processes. Using mixed-effects linear regression, we tested the group effect and its interaction with domains and modalities on the SL composite scores (Table S6). A significant effect of group was observed with the TD group performing significantly better than the ASD group (*b* = 0.33, *SE* = 0.09, *t* = 3.61, *p* < 0.001) (Fig. 2c). We found a significant interaction between group and domain (*b* = 0.35, *SE* = 0.15, *t* = 2.31, *p* = 0.02), reflected by a larger group difference in the linguistic SL tasks than the nonlinguistic SL tasks. Post-hoc analyses in each SL task confirmed that the TD group performed significantly better than the ASD group in both the Letter SL task (*t*(94.06) = 3.67, one-tailed *p* < 0.001) and the Syllable SL task (*t*(86.78) = 2.40, one-tailed *p* = 0.009). To confirm this specific weakness observed in linguistic SL in the ASD group, we further conducted Pearson’s correlations between linguistic SL and nonlinguistic SL composite scores separately in each group. While a marginally significant correlation was found across domains in the TD group (*r*(48) = 0.28, *p* = 0.05), no significant correlation was found in the ASD group (*r*(53) =  − 0.004, *p* = 0.98) (Table S7).

#### Individual Variations in SL Across Domains and Modalities

Using the composite scores, we examined how children’s performance correlates between the SL tasks. In the TD group, performance in Letter SL was significantly correlated with Syllable SL (*r*(38) = 0.48, *p* = 0.002) and with Image SL (*r*(45) = 0.44, *p* = 0.002; Table S7) after corrections for multiple comparisons. In the ASD group, children’s performance in Image SL was marginally correlated with Letter SL (*r*(47) = 0.24, *p* = 0.09) and Tone SL (*r*(43) = 0.29, *p* = 0.06). None of these correlations in the ASD group survived corrections for multiple comparisons. Critically, we compared the significant correlations between groups and the strength of the correlation between Letter SL and Syllable SL was significantly stronger in the TD than the ASD group (*z* = 2.49, *p* = 0.01) (Diedenhofen & Musch, 2015; Fisher, 1925).

### Development of SL in TD vs. ASD

**Fig. 3:**
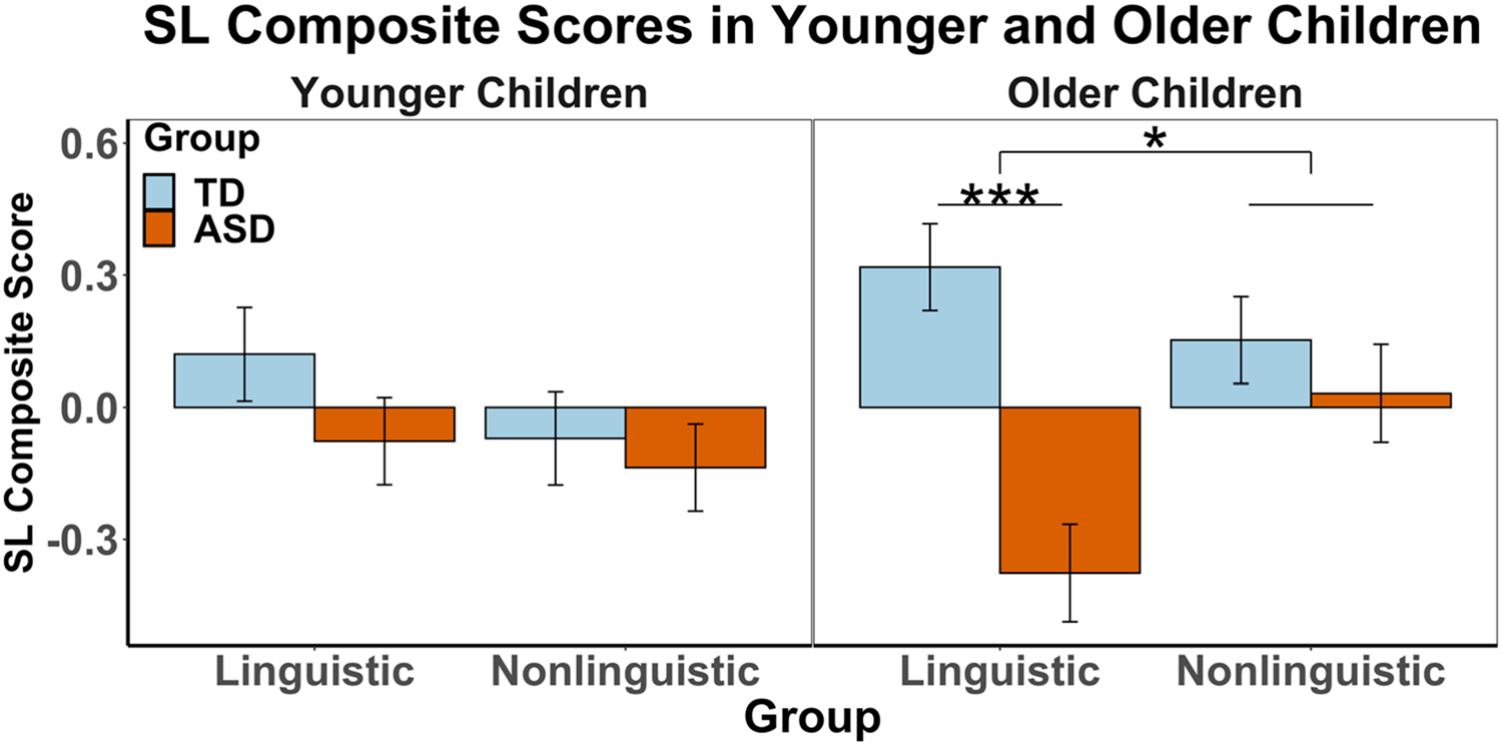
Statistical learning (SL) composite scores in younger and older typically developing (TD) children and children with autism spectrum disorder (ASD). Specific weaknesses in linguistic statistical learning became more evident in older children with ASD. The error bars represent within-group standard error (Morey, 2008). ***p < 0.001, *p < 0.05

We investigated whether the group differences between TD and ASD in SL change over development using two approaches. First, we treated age as a continuous variable and analyzed the interaction between age, group, and domain on the composite SL scores collapsed across sensory modalities (nonlinguistic SL vs. linguistic SL composite scores). Using a mixed-effects linear regression model (Table S8), we found a significant three-way interaction between group, domain, and age (*b* = 0.39, *SE* = 0.16, *t* = 2.43, *p* = 0.02). To follow up on this three-way interaction, we divided the TD and ASD groups into older (Mean Age = 9.65 years; 26 ASD vs. 27 TD) and younger (Mean Age = 7.22 years; 29 ASD vs. 23 TD) subgroups based on a median split on age.^6^ The distribution of language levels (below-age vs. above/at-age) was similar between younger and older children with ASD ($\chi$^2^ = 0.002, *p* = 0.97). About half of the children with ASD in each age group had below-age language level (older ASD: *N* = 12; younger ASD: *N* = 14) as reported by parents. We then analyzed the interaction between diagnosis groups (ASD vs. TD) and domains (linguistic vs. nonlinguistic) on the composite SL scores separately for the younger and older children. Because of the statistically different mean age between TD and ASD within each age group (within the older children: *t*(46.72) = 2.57, *p* = 0.01; within the younger children: *t*(44.27) =  − 2.48, *p* = 0.02), age was entered as a continuous covariate into our mixed-effects linear regression models (Table S9). A significant group by domain interaction was found in older children (*b* = 0.54, *SE* = 0.21, *t* = 2.54, *p* = 0.01), reflected as a larger and significant group difference in the linguistic domain (*t*(43.25) = 3.79, *p* < 0.001) than in the nonlinguistic domain (*p* = 0.50, Fig. 3). However, no significant effect of diagnostic group, domain, or their interaction was found in the younger children.

### Correlation Between SL and Language Measures

**Fig. 4:**
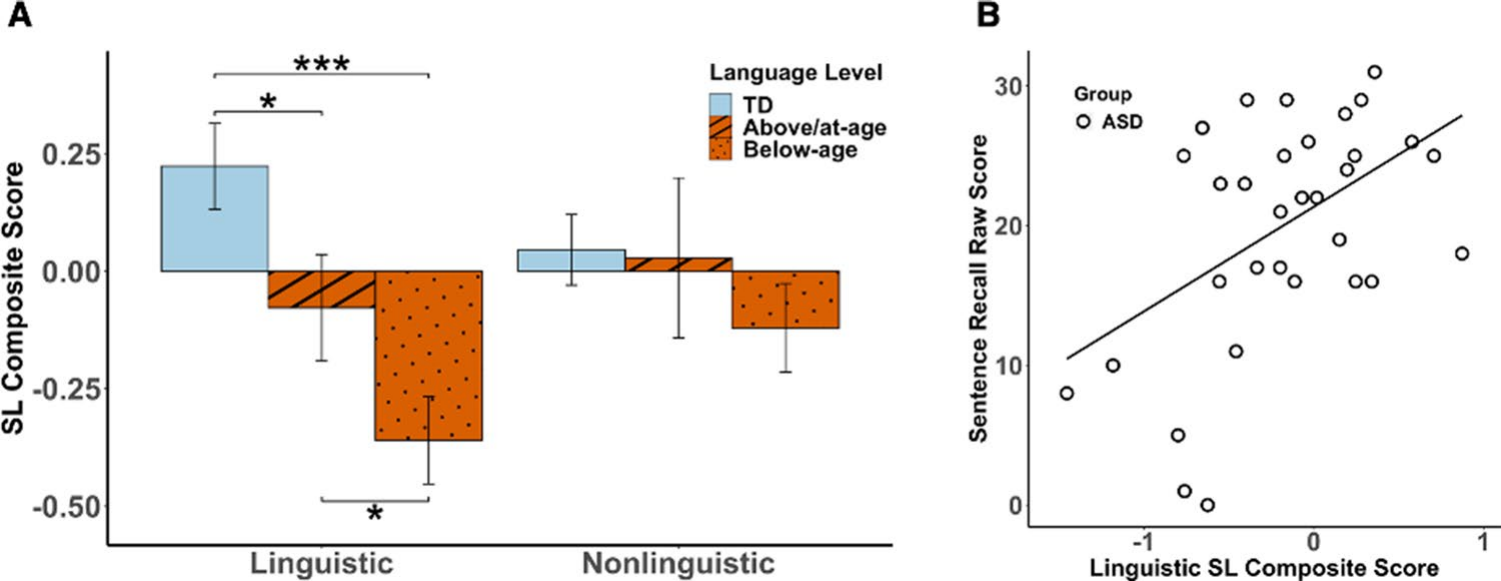
**a** Statistical learning (SL) composite scores in typically developing (TD) children and children with autism spectrum disorder (ASD) with below-age or above/ at-age language levels. Children with ASD across language levels scored lower than TD children in linguistic SL. Children with ASD whose language levels were below age level scored significantly lower than those whose language levels were at or above age level in linguistic SL. **b** Correlation between linguistic SL composite scores and sentence recall raw scores in children with ASD. *p < 0.05, ***p < 0.001

We further examined the correlation between language measures and SL composite scores. In the full sample, children with ASD scored significantly lower than TD children regardless of their language levels in the linguistic SL composite scores (at/above-age language level: *t*(44.58) =  − 2.07, one-tailed *p* = 0.02, g*^7^ = 0.51; below-age language level: *t*(65.48) =  − 4.46, one-tailed *p* < 0.001, g* = 1.02) but not in the nonlinguistic SL (one-tailed *p*’s > 0.08). Children with ASD whose language levels were rated below age level scored significantly lower than those whose language levels were rated at or above age level in the linguistic SL composite scores (*t*(39.62) =  − 1.92, one tailed *p* = 0.03, g* = 0.56) but not in the nonlinguistic SL (one-tailed *p* = 0.22) (Fig. 4a).

In the sample with language assessment scores, we conducted partial Pearson’s correlations between sentence recall raw scores and SL composite scores while controlling for age. A significant partial correlation between linguistic SL composite scores and sentence recall raw scores was found in the ASD group (*r* = 0.57, *p* < 0.001) (Fig. 4b). Similarly, robust correlations were found between sentence recall and the two linguistic SL tasks: Letter SL (*r* = 0.44, *p* = 0.01) and Syllable SL (*r* = 0.44, *p* = 0.02). In contrast, performance in the nonlinguistic SL tasks was not associated with sentence recall scores (see Table S10 for full correlation matrix). Most TD children had ceiling performance in the sentence recall tasks and no significant correlation was found in the TD group.

## Discussion

The current study systematically compared SL between TD children and autistic children across visual and auditory modalities, as well as across linguistic and nonlinguistic domains. Autistic children showed weaknesses in linguistic SL (Syllable and Letter tasks), reflected as slower online learning, poorer offline pattern retrieval, and poorer overall learning performance (measured by composite scores). However, children with autism exhibited comparable performance in nonlinguistic SL (Image and Tone tasks) as their TD peers. The specific weaknesses in linguistic SL appear to be more evident in older children than in younger children with autism. Linguistic SL was further associated with parental subjective ratings of overall language levels in autistic children as well as with standard scores resulting from a standardized language assessment completed by a subgroup of autistic children.

Our findings highlight the asymmetry between linguistic and nonlinguistic SL in children with autism. These results are in concert with most recent theoretical frameworks of SL that emphasize the stimuli-specific computations which operate alongside domain-general learning principals (e.g., Conway, 2020; Frost et al., 2015). Ample empirical evidence in earlier implicit statistical learning literature has demonstrated independent statistical learning systems across auditory and visual sensory modalities (Conway & Christiansen, 2005, 2006; Emberson et al., 2011). The asymmetry and dissociation between linguistic and nonlinguistic SL behavior found in our ASD group further support this view and highlight the operation of stimuli-specific constraints on SL. However, it remains unknown which stages of learning are disrupted in SL in children with autism and why they are specific for the linguistic domain.

Recent theoretical frameworks of SL posit that the learning of statistical information is comprised of multiple stages, including the encoding of individual stimuli from a continuous stream of input, the binding of individual stimuli into word-like units, as well as the storing of these representations for later retrieval (Batterink & Paller, 2017; Bogaerts et al., 2016; Frost et al., 2015). In children with autism, one potential stage of learning that may be disrupted more in linguistic compared to nonlinguistic SL is the initial perceptual processing stage where individual stimuli are encoded from sequential inputs. Existing behavioral research has indicated that children with autism show specific difficulties in processing auditory linguistic input as manifested in reduced orientation to speech, such as human voice and child-directed speech, compared to non-speech sounds (Dawson et al., 1998, 2004; Kuhl et al., 2005; see O’Connor, 2012 for a review). Neurophysiological data also suggest more diminished neural responses to speech syllables during passive listening in autistic children compared to TD controls (Jansson-Verkasalo et al., 2003; Russo et al., 2009). One prominent theory is the neural complexity hypothesis, which posits that children with autism exhibit more salient difficulties in processing complex over simple auditory and visual stimuli (Bertone et al., 2005; Mottron et al., 2006; Samson et al., 2006). Speech sounds, for example, contain greater spectro-temporal complexity compared to pure tones, thus reduced sensitivity to speech sounds in children with autism may impact learning in the syllable SL task. In our study, however, we found little evidence in the disadvantage of sensitivity across the linguistic and nonlinguistic domains in children with autism. Children’s accuracy in target detection based on their *A’* scores did not affect the group differences in RT slopes or the interaction between group and domain. One possibility is that the target detection task we adopted is not sensitive enough to capture children’s baseline orientation or sensitivity to linguistic versus nonlinguistic stimuli. Future studies including tasks comparing the complexity of auditory and visual stimuli are necessary to delineate the role of perceptual processing in SL.

Another plausible cause for the difficulties in SL may lie at the higher level of statistical learning where the binding of individual stimuli and computations of the relationships between stimuli must occur for the extraction of statistical regularities in the inputs. In neurotypical adults, the ability to seek out and track probabilistic relationships between individual stimuli were found to predict SL performance (Batterink & Paller, 2017). In school-aged children with ASD, however, reduced neural sensitivity to statistical regularities embedded in verbal stimuli has been reported using a syllable SL task (Scott-Van Zeeland et al., 2010). In this study, children were listening passively to syllable streams containing embedded triplet structures versus those containing random sequences. Unlike TD children who showed greater brain activation in response to the structured than random sequences, autistic children showed no difference between the two conditions. In our study, despite the evident difficulties in the ASD group measured by composite linguistic SL scores, the group differences in the two linguistic SL tasks appear at different phases of the SL tasks. Children with ASD showed poorer retrieval of triplets after learning in both the Letter and Syllable SL tasks, and also showed slower target detection during learning in the Letter SL task. Slower target detection during learning may stem from difficulties with encoding and binding linguistic stimuli, computing probabilistic relationships, or learning the predictive parings between stimuli (Cannon et al., 2021). This less robust learning process leads to a weaker representation and poorer retrieval of statistical regularities from memory. The neural networks that underlie these difficulties may be impacted, such as the lower- or higher-level visual and auditory networks supporting the stimulus-specific processing mechanisms or the medial-temporal lobe supporting domain-general memory systems (Frost et al., 2015; Schapiro et al., 2014). Although the current study cannot distinguish between these accounts or pin down the underlying cause of atypical linguistic SL in children with ASD, future studies can address the gap by examining the neural profiles of SL in ASD.

In addition to the specific differences in linguistic SL tasks, children with ASD also showed poorer retrieval of triplets in the Image SL task compared to TD children, while both groups showed a lack of acceleration during online image target detection. The lack of group-level acceleration might be explained by the large individual differences in both SL and perceptual acuity. But importantly, there was no apparent disadvantage of image SL in ASD as measured by online RT slope. The reduced 2AFC accuracy in ASD may indicate a pronounced difficulty in this post-learning task that requires the retrieval of image patterns. Prior work that examined children’s sensitivity to transitional probabilities in a nonlinguistic visual SL task also found reduced attention and visual discrimination to pairs of shapes in the post-learning test phase in young children with ASD compared to TD peers (Jeste et al., 2015). Retrieving image patterns may tax visual working memory. Individuals with ASD have been found to show atypical visual working memory (Baum et al., 2015; Funabiki & Shiwa, 2018; Stevenson et al., 2021). Whether differences in visual working memory can explain the reduced 2AFC accuracy after image SL in autism would require further investigation.

Lastly, our findings support the reciprocal relationship between language experiences and language learning. Enriched language experiences facilitate linguistic SL in adults (Potter et al., 2017; Siegelman et al., 2018a; Wang & Saffran, 2014). In children, we found a significantly stronger correlation between children’s performance in the two linguistic SL tasks in the TD group than that in the ASD group. This may suggest the learning of statistical regularities embedded in speech and written language are meaningfully coupled in typical development while showing a disassociation in children with autism. This coupling of linguistic SL in the TD group might be explained by the commonly observed mutual development of phonological and decoding skills in school-aged TD children (see Hulme & Snowling, 2014 for a review), while the weaker correlation in the ASD group might be explained by the disassociation between oral language and reading skills in children with ASD (Macdonald et al., 2021; Smith Gabig, 2010). However, the current study cannot determine the causal relationship between language experience and learning. Whether the mutual development of spoken and written language supports the association in linguistic SL or vice versa warrants further investigations. Furthermore, we found a significantly larger diagnostic group difference in linguistic SL compared to nonlinguistic SL in the older children. Our findings suggest a potentially exacerbating difficulty in linguistic SL in older children compared to younger children with ASD. The larger gap between ASD and TD groups in linguistic SL found only in older children but not in younger children is consistent with previous findings that demonstrated the impact of prior language experience on linguistic SL (Saffran & Kirkham, 2018). Older TD children may have greater language experience compared to age-matched children with ASD, and the enlarged gap in language experience throughout development may exacerbate the difficulty in linguistic SL observed in older children with ASD. Longitudinal datasets will be indispensable to unravel the causal relationship between language experience and linguistic SL development.

The reciprocal relationship between language and linguistic SL was further strengthened by the association between sentence recall scores and linguistic SL composite scores. The significant associations found between language skills measured by the sentence recall task and linguistic SL, Letter, and Syllable SL tasks in the ASD group may imply the contribution of SL to language. Our findings are consistent with the significant correlations between word segmentation performance and language skills reported in Haebig et al. (2017), where language skills were measured by Peabody Picture Vocabulary Test-4 (PPVT-4; Dunn & Dunn, 2007) and the Clinical Evaluation of Language Fudamentals-4 (CELF-4; Semel et al., 2003) core language scores. While the current dataset serves as a promising first step implicating the potential value of linguistic SL in characterizing children’s language learning profiles, rigorous psychometric evaluations are important to further validate the composite score as a reliable individual difference measure across typical and atypical development. Our findings also measured only concurrent language skills with one standardized language assessment. Given the large heterogeneity across different constructs of language in autism, future longitudinal research are necessary to verify whether SL has cascading effects on all domains of language development and whether different types of language experience, in turn, lead to differences in linguistic SL.

Our findings also suggest a parallel impact of autism on linguistic SL that might be separate from the interplay between language skills and linguistic SL. Using the parental report of language levels, we found the subgroup of autistic children with at/above-age language outperformed the subgroup with below-age language specifically in the linguistic SL tasks, implicating linguistic SL is perhaps a relevant indicator of language phenotypes in autism (Lucas & Norbury, 2014; McGregor et al., 2012; Norbury, 2014). Importantly, we also found children with ASD across language levels showed poorer lingusitic SL than TD children, suggesting autism, in addition to language skills, might be associated with the ability to learn probabilistic and predictive relationships between inputs (Cannon et al., 2021; Sinha et al., 2014). This finding emphasizes that multiple causes might underlie the differences observed in linguistic SL between the TD and ASD groups. However, it is important to point out that, while the subgroup of autistic children with at/above-age language had higher sentence recall scores than the subgroup with below-age language, parental report of language levels may still be a coarse measure of overall language skills. Future investigations including a sample of autistic children with typical language as well as non-autistic children with atypical language measured by a comprehensive battery of language assessments will be needed to elucidate the relationships between autism, language, and SL.

In sum, children with autism exhibit specific differences in linguistic SL and such differences might be attributed to a more pronounced difficulty in perceptual processing of linguistic than nonlinguistic stimuli, reduced sensitivity to statistical regularities, or an exacerbating gap in linguistic experiences across development. These findings highlight the potential role of linguistic SL in the characterization of language development in autism and provide important clinical implications for remediating language impairment in children with autism. The current study only measured language skills using parental ratings and one standardized language asssessment. Future studies including more comprehensive language measures would be needed to examine whether SL contributes differentially to different aspects of language. If SL and some aspect of language develop in a virtuous cycle—that is, SL can facilitate language learning, and language can in turn support SL—then linguistic SL might serve as an intervention tool to prevent the snowballing process for atypical language development in autism. Behavioral research has suggested that SL might be malleable. For example, infants with prior exposure to distributional information in speech, such as adjacent co-occurrence patterns and prosodic cues, were more likely to learn novel and more complex statistical patterns from subsequent speech than those without prior exposure (Lany & Gómez, 2008; Thiessen & Saffran, 2007). Further studies investigating the malleability of SL within individuals and determining which SL processes might be sensitive to intervention and cascade into improved language learning will enable us to develop novel effective interventions for autistic children.

### Supplementary Information

Below is the link to the electronic supplementary material.

Supplementary file1 (DOCX 39 kb)

## Data Availability

The datasets used and analysed during the current are available in Open Science Framework (OSF) repository, https://osf.io/4k7mx/.

## Footnotes

1 Recent research shows that many, but not all, members of the autistic community prefer identity-first language (“autistic person”) to person-first language (“person with autism”) (Lei et al., 2021). We chose to alternate between identify-first and person-first language throughout the manuscript to honor this preference.

2 One child with ASD was missing the source of diagnosis from SPARK.

3 The parental reports of language level were obtained from SPARK database. Nine children with ASD were missing parental reports of language level from SPARK.

4 Mean Sentence Recall raw scores: at- or above-age level: 23.77; below age level: 15.13. Mean age-normed standard scores: at- or above-age level: 91.70; below age level: 70.57.

5 *A’* = 1/2 ± (*hit* − *fa*) * (1 + *hit* − *fa*)/ 4 * *hit* * (1 − *fa*), where *hit* represents correct identifications of the target stimuli and *fa* (false alarms) represents incorrect responses to stimuli that are not the target. A higher *A’* indicates better discriminability (Pallier, 2002).

6 Mean age of older TD: 10.00; older ASD: 9.23; younger TD: 6.96; younger ASD: 7.42 years.

7 Hedge’s *g** was used to calculate the effect size of the Welch’s *t*-tests conducted (Delacre et al., 2021).
