# Supplementary material for: Dissociation Between Linguistic and Nonlinguistic Statistical Learning in Children with Autism
Source: J Autism Dev Disord. 2023 Feb 7;54(5):1912–27. doi: 10.1007/s10803-023-05902-1 (PMC10404646; doi:10.1007/s10803-023-05902-1)
Supplement: Supplementary file 1 — Supplementary file1 (DOCX 39 kb) [file 10803_2023_5902_MOESM1_ESM.docx]

**Supplementary Material**

**Table S1**.

Total number of participants included in the statistical learning (SL) tasks

|  | **SL Tasks** | **TD** | **ASD** |
| --- | --- | --- | --- |
| **Reaction Time Slope** | **Letter** | 46 | 50 |
|  | **Syllable** | 43 | 48 |
|  | **Image** | 48 | 49 |
|  | **Tone** | 44 | 46 |
| **Accuracy** | **Letter** | 47 | 52 |
|  | **Syllable** | 43 | 51 |
|  | **Image** | 50 | 52 |
|  | **Tone** | 45 | 48 |

**Table S2**.

Mean number of valid responses during the familiarization phase of the SL tasks

|  | SL Tasks | **TD** | **ASD** | **Group Comparison** |
| --- | --- | --- | --- | --- |
| **Hits** | **Letter** | 22.43 [21.86, 23.01] | 21.48 [20.55, 22.41] | *t*(80.94) = 1.75, *p* = 0.08 |
|  | **Syllable** | 27.67 [24.24, 31.11] | 28.25 [25.27, 31.23] | *t*(85.80) = -0.26, *p* = 0.80 |
|  | **Image** | 22.46 [21.88, 23.03] | 22.18 [21.62, 22.75] | *t*(94.95) = 0.68, *p* = 0.50 |
|  | **Tone** | 23.59 [20.37, 26.81] | 24.22 [21.17, 27.26] | *t*(87.48) = -0.28, *p* = 0.78 |
| **A-prime** | **Letter** | 0.98 [0.97, 0.99] | 0.97 [0.96, 0.98] | *t*(76.46) = 2.02, *p* = 0.05 |
|  | **Syllable** | 0.86 [0.83, 0.88] | 0.84 [0.82, 0.87] | *t*(84.42) = 0.76, *p* = 0.45 |
|  | **Image** | 0.98 [0.97, 0.99] | 0.98 [0.97, 0.98] | *t*(94.95) = 0.90, *p* = 0.37 |
|  | **Tone** | 0.78 [0.75, 0.81] | 0.77 [0.74, 0.79] | *t*(80.64) = 0.75, *p* = 0.46 |

95% confidence interval is reported in square brackets.

**Table** **S3**.

Mean reaction time slope, accuracy, and composite scores in TD children and children with ASD

| **SL Task** | **Reaction Time Slope** | | **Accuracy** | | **Composite Score** | |
| --- | --- | --- | --- | --- | --- | --- |
|  | **TD** | **ASD** | **TD** | **ASD** | **TD** | **ASD** |
| **Letter** | -0.02  [-0.03, -0.007] | 0.004  [-0.009, 0.02] | 0.62  [0.57, 0.66] | 0.52  [0.48, 0.56] | 0.29  [0.07, 0.51] | -0.26  [-0.46, -0.07] |
| **Syllable** | -0.02  [-0.02, -0.01] | -0.01  [-0.02, -0.002] | 0.57  [0.53, 0.60] | 0.52  [0.50, 0.54] | 0.18  [-0.03, 0.39] | -0.17  [-0.35, 0.01] |
| **Image** | 0.006  [-0.006, 0.02] | -0.003  [-0.02, 0.009] | 0.66  [0.60, 0.71] | 0.56  [0.52, 0.60] | 0.08  [-0.14, 0.31] | -0.09  [-0.27 0.10] |
| **Tone** | -0.002  [-0.006, 0.003] | -0.003  [-0.009, 0.002] | 0.58  [0.54, 0.62] | 0.56  [0.53, 0.59] | 0.006  [-0.19, 0.20] | -0.04  [-0.26, 0.17] |

95% confidence interval is reported in square bracket

**Table S4**.

Online Learning (reaction time analysis): Fixed effects of the ﻿mixed-effects linear regression model (total observations: 8969)

|  | *b* | *SE* | *t* | *p* |
| --- | --- | --- | --- | --- |
| (Intercept) | 0.11 | 0.02 | 4.98 | < 0.001*** |
| Group | 0.08 | 0.04 | 1.81 | 0.07^†^ |
| Trial order | -0.006 | 0.001 | -4.69 | < 0.001*** |
| Domain | 0.16 | 0.04 | 3.85 | < 0.001*** |
| Modality | -0.10 | 0.04 | -2.28 | 0.02* |
| Gender | 0.001 | 0.02 | 0.04 | 0.96 |
| Group:Trial order | -0.004 | 0.002 | -1.80 | 0.07^†^ |
| Group:Domain | 0.25 | 0.08 | 2.96 | 0.003** |
| Trial order:Domain | -0.01 | 0.002 | -3.94 | < 0.001*** |
| Group:Modality | -0.05 | 0.08 | -0.61 | 0.54 |
| Trial order:Modality | 0.002 | 0.002 | 0.79 | 0.43 |
| Domain:Modality | -0.07 | 0.08 | -0.80 | 0.42 |
| Group:Trial order:Domain | -0.02 | 0.005 | -3.65 | < 0.001*** |
| Group:Trial order:Modality | 0.0002 | 0.005 | 0.05 | 0.96 |
| Group:Domain:Modality | 0.22 | 0.17 | 1.34 | 0.18 |
| Trial Order:Domain:Modality | -0.002 | 0.005 | -0.37 | 0.71 |
| Group:Trial order:Domain:Modality | -0.02 | 0.01 | -2.43 | 0.02* |

Group = ASD (-0.5) vs. TD (0.5); Trial order = 1 to 24 in visual tasks or 1 to 48 in auditory tasks; Domain = nonlinguistic (-0.5) vs. linguistic (0.5); Modality = auditory (-0.5) vs. visual (0.5); Gender = Female (-0.5) vs. Male (0.5). ^†^*p* = 0.07; **p* < 0.05; ***p* < 0.01; ****p* < 0.001.

**Table S5**.

Offline Learning (accuracy analysis): Fixed effects of the ﻿ mixed-effects logistic regression model (total observations: 12416)

|  | *b* | *SE* | *z* | *p* |
| --- | --- | --- | --- | --- |
| (Intercept) | 0.32 | 0.05 | 7.11 | < 0.001*** |
| Group | 0.32 | 0.09 | 3.45 | < 0.001*** |
| Domain | -0.16 | 0.05 | -3.10 | 0.002** |
| Modality | 0.19 | 0.07 | 2.60 | 0.009** |
| Gender | 0.04 | 0.08 | 0.47 | 0.64 |
| Group:Domain | 0.06 | 0.11 | 0.54 | 0.59 |
| Group:Modality | 0.34 | 0.14 | 2.34 | 0.02* |
| Domain:Modality | -0.10 | 0.08 | -1.24 | 0.22 |
| Group:Domain:Modality | -0.12 | 0.16 | -0.77 | 0.44 |

Group = ASD (-0.5) vs. TD (0.5); Domain = nonlinguistic (-0.5) vs. linguistic (0.5); Modality = auditory (-0.5) vs. visual (0.5); Gender = Female (-0.5) vs. Male (0.5). **p* < 0.05; ***p* < 0.01; ****p* < 0.001.

**Table S6**.

Composite scores analysis: Fixed effects of the ﻿mixed-effects linear regression model

(total observations: 388)

|  | *b* | *SE* | *t* | *p* |
| --- | --- | --- | --- | --- |
| (Intercept) | -0.02 | 0.04 | -0.4 | 0.69 |
| Group | 0.33 | 0.09 | 3.61 | < 0.001*** |
| Domain | 0.01 | 0.07 | 0.18 | 0.86 |
| Modality | 0.01 | 0.07 | 0.14 | 0.89 |
| Gender | 0.12 | 0.09 | 1.36 | 0.18 |
| Group:Domain | 0.35 | 0.15 | 2.31 | 0.02* |
| Group:Modality | 0.16 | 0.14 | 1.11 | 0.27 |
| Domain:Modality | -0.01 | 0.13 | -0.11 | 0.91 |
| Group:Domain:Modality | 0.09 | 0.26 | 0.36 | 0.72 |

Group = ASD (-0.5) vs. TD (0.5); Domain = nonlinguistic (-0.5) vs. linguistic (0.5); Modality = auditory (-0.5) vs. visual (0.5); Gender = Female (-0.5) vs. Male (0.5). **p* < 0.05; ****p* < 0.001.

**Table S7.**

Pearson’s Correlation Matrix of SL Composite Scores across Tasks

|  | **TD** | | | | **ASD** | | | | |
| --- | --- | --- | --- | --- | --- | --- | --- | --- | --- |
| **SL tasks** | **Syllable** | **Image** | **Tone** | **Linguistic** | | **Syllable** | **Image** | **Tone** | **Linguistic** |
| **Letter** | 0.48** | 0.44** | 0.16 |  | | -0.03 | 0.24^†^ | 0.04 |  |
| **Syllable** |  | 0.13 | -0.04 |  | |  | -0.20 | -0.24 |  |
| **Image** |  |  | 0.04 |  | |  |  | 0.29^†^ |  |
| **Nonlinguistic** |  |  |  | 0.28^†^ | |  |  |  | -0.004 |

Underlined correlations remained significant after Bonferroni corrections for multiple comparisons. ^†^*p* < 0.1, ***p* < 0.01.

**Table S8**.

Development analysis: Fixed effects of the mixed-effects linear regression model

(total observations: 210)

|  | *b* | *SE* | *t* | *p* |
| --- | --- | --- | --- | --- |
| (Intercept) | -0.001 | 0.04 | -0.03 | 0.98 |
| Group | 0.25 | 0.08 | 2.97 | 0.004** |
| Domain | -0.02 | 0.07 | -0.23 | 0.82 |
| Age | 0.10 | 0.05 | 2.08 | 0.04* |
| Group:Domain | 0.37 | 0.15 | 2.54 | 0.01* |
| Group:Age | -0.03 | 0.09 | -0.34 | 0.74 |
| Domain:Age | -0.13 | 0.08 | -1.60 | 0.11 |
| Group:Domain:Age | 0.39 | 0.16 | 2.43 | 0.02* |

Group = ASD (-0.5) vs. TD (0.5); Domain = nonlinguistic (-0.5) vs. linguistic (0.5). **p* < 0.05; ***p* < 0.01.

**Table S9**.

Development analysis based on a median-split on age: Fixed effects of the﻿ mixed-effects linear regression model in the younger (S8A) and older (S8B) children

S9A. Younger children (total observations: 104):

|  | *b* | *SE* | *t* | *p* |
| --- | --- | --- | --- | --- |
| (Intercept) | -0.06 | 0.05 | -1.06 | 0.30 |
| Group | 0.18 | 0.11 | 1.62 | 0.11 |
| Domain | 0.13 | 0.10 | 1.26 | 0.22 |
| Age | 0.05 | 0.06 | 0.94 | 0.35 |
| Group:Domain | 0.19 | 0.21 | 0.91 | 0.37 |

S9B. Older children (total observations: 106):

|  | *b* | *SE* | *t* | *p* |
| --- | --- | --- | --- | --- |
| (Intercept) | 0.05 | 0.07 | 0.69 | 0.49 |
| Group | 0.33 | 0.14 | 2.34 | 0.02* |
| Domain | -0.13 | 0.11 | -1.22 | 0.23 |
| Age | 0.08 | 0.07 | 1.20 | 0.24 |
| Group:Domain | 0.54 | 0.21 | 2.54 | 0.01* |

Group = ASD (-0.5) vs. TD (0.5); Domain = nonlinguistic (-0.5) vs. linguistic (0.5). **p* < 0.05.

**Table S10**.

Partial Pearson’s Correlations between SL Composite Scores and Sentence Recall Raw Scores after controlling for age.

| **Group** | **Letter** | **Syllable** | **Image** | **Tone** | **Linguistic** | **Nonlinguistic** |
| --- | --- | --- | --- | --- | --- | --- |
| **TD** | -0.0003 | 0.01 | -0.11 | 0.14 | -0.02 | -0.08 |
| **ASD** | 0.44* | 0.44* | 0.23 | 0.10 | 0.57*** | 0.23 |

Underlined correlations remained significant after Bonferroni corrections for multiple comparisons. ^*^*p* < 0.05; ****p* < 0.001.
